# Effects of Transient Hypoxia versus Prolonged Hypoxia on Satellite Cell Proliferation and Differentiation In Vivo

**DOI:** 10.1155/2015/961307

**Published:** 2015-02-18

**Authors:** Sukanta Jash, Samit Adhya

**Affiliations:** ^1^Molecular and Human Genetics Division, CSIR-Indian Institute of Chemical Biology, 4 Raja S. C. Mullick Road, Kolkata 700032, India; ^2^Center for Regenerative Medicine, Massachusetts General Hospital, Boston, MA 02114, USA

## Abstract

The microenvironment of the injury site can have profound effects on wound healing. Muscle injury results in ischemia leading to short-term local hypoxia, but there are conflicting reports on the role of hypoxia on the myogenic program in vivo and in vitro. In our rat model of mitochondrial restoration (MR), temporary upregulation of mitochondrial activity by a cocktail of organelle-encoded RNAs results in satellite cell proliferation and initiation of myogenesis. We now report that MR leads to a transient hypoxic response in situ. Inhibition of hypoxia by lowering mitochondrial O_2_ consumption, either by respiratory electron transport inhibitors, or by NO-mediated inhibition of O_2_ binding to cytochrome c oxidase, resulted in exacerbation of inflammation. Lentivirus-mediated knockdown of hypoxia-inducible factor 1*α* (HIF1*α*) or of Notch signaling components had a similar effect, and pharmacologic inhibition of HIF or Notch reduced the number of proliferating Pax7^+^ cells. In contrast, a prolonged hypoxic response induced either by uncoupling of respiration from oxidative phosphorylation or through HIF stabilization by dimethyloxalylglycine (DMOG) had an immediate anti-inflammatory effect. Although significant satellite cell proliferation occurred in presence of DMOG, expression of differentiation markers was affected. These results emphasize the importance of transient hypoxia as opposed to prolonged hypoxia for myogenesis.

## 1. Introduction

Regeneration of adult mammalian muscle postinjury is a complex process involving tightly regulated molecular events that follow an initial inflammatory response, including infiltration of immune cells and clearance of cellular debris at the injury site, activation and proliferation of satellite cells (SCs) to form myoblasts, and their subsequent differentiation and fusion to form myofibers [1]. In parallel, angiogenesis with restoration of blood supply occurs, and neural connectivity is restored. The process of satellite cell activation occurs in this continuously changing microenvironment of the injury site.

One of the consequences of muscle injury caused by burn, laceration, or freezing is the rupture of blood vessels resulting in ischemia and lowering of local O_2_ concentration. Local hypoxia has been observed at the injury site after incision, with gradual rise of O_2_ levels after 4–10 days [2–4], but its role, if any, in myogenesis in vivo is unclear. Under continuous hypobaric hypoxia, the recovery of muscle mass through regeneration was temporarily delayed [5]. Prolonged exposure of cultured myoblasts to hypoxic conditions has been reported to block their differentiation [6]; accumulation of Pax7^+^MyoD^−^ cells and decline of Pax7^+^MyoD^+^ cells, without an overall effect on myoblast proliferation under hypoxia, have been reported [7]. On the other hand, the proliferation of cloned SCs is apparently stimulated by hypoxia [8]. Tissue-specific knockout (KD) of HIF1*α* in muscle cells did not affect cellular proliferation, but KD of HIF1*α* in myeloid cells resulted in reduced macrophage infiltration and numbers of proliferating cells [9], suggesting that hypoxia indirectly triggers myoblast proliferation through phagocytic clearance of the debris at the injury site. Thus, a number of outstanding questions are yet to be answered: (1) whether hypoxia directly affects satellite cell proliferation or merely the initial inflammatory response and (2) since wound hypoxia is transient, whether the duration of hypoxia has any specific effect.

We have previously reported that restoration of mitochondrial activity at the injury site of rat muscle by administration of a cocktail of mitochondrion-targeted RNAs remarkably accelerates satellite cell activation and initiation of the myogenic program [10]. Since, in our current protocol, mitochondrial restoration (MR) is induced at the peak of inflammation, regenerative processes may be distinguished from earlier inflammatory events. Moreover, due to degradation of the RNAs within mitochondria, MR in normal adult muscle is transient, with a peak of mitochondrial oxidative capacity at ~6 h [11]; thus, transient MR may act as a trigger of SC activation. We have now examined the effect of MR on tissue hypoxia and regeneration. We report that whereas MR-induced transient hypoxia stimulates SC proliferation followed by differentiation, conditions that inhibit hypoxia increase inflammation, but prolonging the hypoxic response has an adverse effect on myoblast differentiation.

## 2. Materials and Methods

### 2.1. Regeneration Model

Sprague-Dawley rats received needle injury in the hind limb quadriceps muscle. Lesion circumference was measured daily. At the height of inflammation (6 d after injury), a cocktail of three polycistronic RNAs encoding various portions of the rat mitochondrial genome, or control D arm oligonucleotide, was administered at the injury site as ribonucleoprotein (RNP) complexes with RNA import complex (RIC), as described [10].

### 2.2. In Situ Detection of Hypoxia

Following administration of pcRNAs, 0.9% Hypoxyprobe-1 (Pimonidazole Hydrochloride; Hypoxyprobe, Inc.) in PBS was injected intraperitoneally at a dosage of 60 mg/kg body weight. After 60 min, the animals were sacrificed and the muscle was excised and immediately fixed with paraformaldehyde. Sections were stained with FITC-conjugated monoclonal antibody against pimonidazole (1 : 500) and DAPI or anti-COII antibody, for confocal microscopy.

### 2.3. FACS Analysis

Mononuclear cells isolated from regenerating muscle were analyzed for Pax7^+^ satellite cells by FACS as described [10].

### 2.4. Inhibitors

HIF inhibitor (HIF-In, Merck-Calbiochem, 3-(2-(4-adamantan-1-yl-phenoxy)-acetylamino)-4-hydroxybenzoic acid methyl ester) specifically prevents hypoxia-induced upregulation of HIF1*α* protein [12]. Dimethyloxalylglycine (DMOG, Sigma) is an inhibitor of prolyl hydroxylase [13]. *γ*-Secretase inhibitor XX (Merck-Calbiochem) inhibits processing of Notch protein to active NICD [14]. Diethylenetriamine/nitric oxide adduct (DETA/NO) releases NO into solution. Inhibitor (100 *μ*L of 2 mM HIF-In, 2 mM DMOG, 100 *μ*M *γ*-secretase inhibitor, or 1 mM DETA-NO) was injected into the wound site 4 h after pcRNA administration. Chloramphenicol (100 *μ*g/mL),* m*-chlorocarbonylcyanide phenylhydrazone (CCCP, 100 *μ*M), or antimycin A (AntA, 100 *μ*M) was injected in 0.1 mL at the wound site 3 h following administration of pcRNA and the muscle was harvested after a further 0.5–1 h.

### 2.5. Lentivirus-Mediated Knockdown

At 3 d and 4 d after injury, the injury site was infected with lentivirus pLKO.1-CMV-tGFP (>4.9 × 10^6^ TU/mL; Sigma Aldrich; thrice daily) expressing nontargeted or gene-targeted short hairpin (sh) RNA (see Supplementary Table S1 available online at http://dx.doi.org/10.1155/2015/961307).

### 2.6. Oxygen Uptake

The rate of O_2_ consumption by excised, permeabilized regenerating muscle was measured using a Clarke-type oxygen electrode (Hansatech Oxygraph).

### 2.7. Imaging

Muscle sections (10 *μ*m) were fixed, incubated with the appropriate primary (Supplementary Table S2) and Alexa Fluor (AF) 395 (blue), AF 488 (green), or AF 633 (red) labeled secondary antibody (1 : 1000), and imaged with the Nikon A1R confocal microscope using the corresponding laser lines.

### 2.8. Western Blot

Blots of muscle protein were probed with the appropriate antibody (Supplementary Table S2) and signals detected by enhanced chemiluminescence.

### 2.9. Quantitative RT-PCR

Muscle RNA was reverse-transcribed using antisense cyclin D1 (Ccnd1) primer, and the cDNA subjected to real-time PCR in presence of SYBR Green in the StepOnePlus (Applied Biosystems) thermal cycler, for 40 cycles with a 45 s annealing step at 55°C, using sense and antisense rat Ccnd1 primers (Supplementary Table S3). The use of gene-specific primers for cDNA synthesis was preferred over synthesis of total cDNA by random priming since the latter yields cDNAs of variable length and sequence coverage from any particular mRNA, only a fraction of which would be amplified by gene-specific primers in the PCR step. mRNA was quantified as fold-change between pcRNA-treated samples and controls; fold-change = 2^−ΔΔC_t_^, where ΔC_t_ = C_t_ (target gene) − C_t_ (*β*-actin), C_t_ is the critical threshold of the SYBR Green signal, and *β*-actin is the internal control.

## 3. Results

### 3.1. Hypoxic Response of pcRNA-Treated Injured Muscle

**Figure 1:**
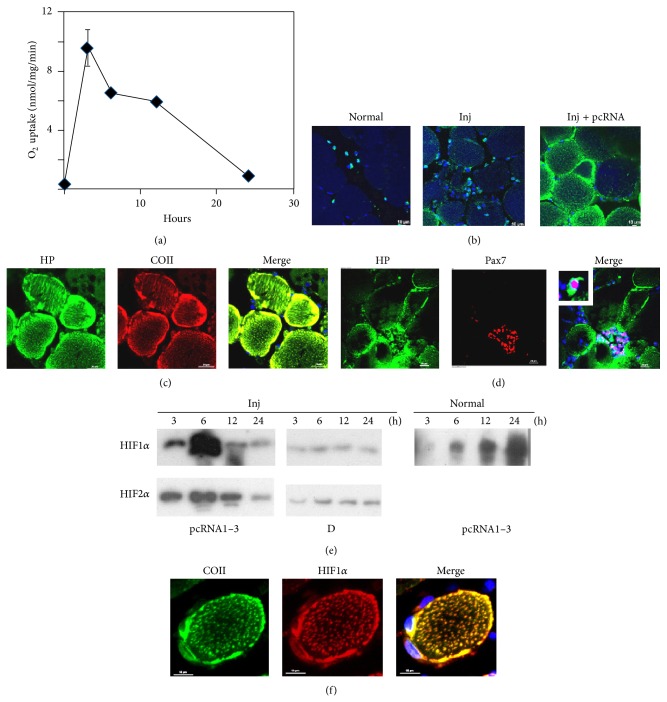
The hypoxic response at the injury site after mitochondrial restoration. (a) O_2_ uptake rate (state 3 respiration) of mitochondria from 6 d injured muscle treated with the pcRNA1–3 combination for the indicated times. (b) Normal (left), injured (middle), or injured muscle treated with pcRNA1–3 for 4 h (right). Hypoxyprobe was injected at the injury site, and the tissue was fixed after 1 h and stained with FITC-labeled antipimonidazole antibody. (c, d) Injured muscle treated with pcRNA1–3 was double stained for Hypoxyprobe (green) and COII (red, (c)) or Pax7 (red, (d)). (e) Western blots of injured (inj) or normal muscle treated with pcRNA1–3, or control D arm, for the indicated times, probed with anti-HIF1*α* (upper row) or HIF2*α* (lower row). (f) A higher magnification image of a single myofiber after pcRNA treatment for 3 h, showing colocalization of HIF1*α* (red) with mitochondria expressing COII (green). Scale bars, 10 *μ*m.

The O_2_ uptake rate of injured muscle of 12–14-month-old rats was reduced to less than 5% of untreated controls (data not shown; see [10]). Administration of control (D arm) RNA had no effect on this basal level; however, in injured muscle treated with the pangenomic pcRNA cocktail, there was a rapid (3 h) and robust (300-fold) induction of respiratory capacity to greater than normal levels (Figure 1(a)). The respiratory capacity peaked at ~3 h but declined to ~4-fold the basal value after 24 h (Figure 1(a)), emphasizing the transient nature of MR.

Hypoxia in pcRNA-treated injured muscle in situ was detected as intracellular adducts of pimonidazole (Hypoxyprobe), the formation of which is detectable only at O_2_ tensions below 10 mm Hg [15]. In quadriceps muscle of normal or injured rats, there was background staining of the myofibers, with a few mononuclear cells containing cytoplasmic deposits of the stain (Figure 1(b)). Treatment of injured muscle with pcRNA1–3 for 4 h resulted in intense particulate staining of the intact myofibers at the injury site, especially near the plasma lemma (Figure 1(b)). The fluorescent adducts colocalized with COII, demonstrating hypoxia at mitochondrial loci (Figure 1(c)). Clusters of Pax7^+^ SCs with intense cytoplasmic Hypoxyprobe stain were observed within hypoxic areas between or close to myofibers, while SCs at some distance from these areas were not stained (Figure 1(d)), suggesting that mitochondrial activity in the myofibers creates a hypoxic microenvironment for proliferation of SCs.

Western blot analysis of control injured muscle treated with a mitochondrion-targeted RNA that has no effect on mitochondrial translation (the D arm of tRNA^Tyr^ [10]) showed that the level of hypoxia inducible factor alpha (HIF1*α* and 2*α*) remained low for at least 24 h (Figure 1(e)). Upon MR, HIF levels went up sharply between 3 and 6 h, declining subsequently (Figure 1(e)). In normal, uninjured muscle treated with pcRNAs, low levels of HIF*α* appeared at 6 h and gradually peaked towards 24 h (Figure 1(e)). In individual myofibers at the injury site, there was accumulation of HIF1*α* at or near the activated mitochondria (Figure 1(f)). These data indicate that a combination of mitochondrial restart and the ischemic environment of the injured tissue generates acute hypoxia, resulting in transient stabilization of HIF subunits through mitochondrion-proximal inhibition of the O_2_ sensor prolyl hydroxylase (PHD) [16].

### 3.2. Effect of Perturbation of Local O_2_ Concentration on Regeneration

**Figure 2:**
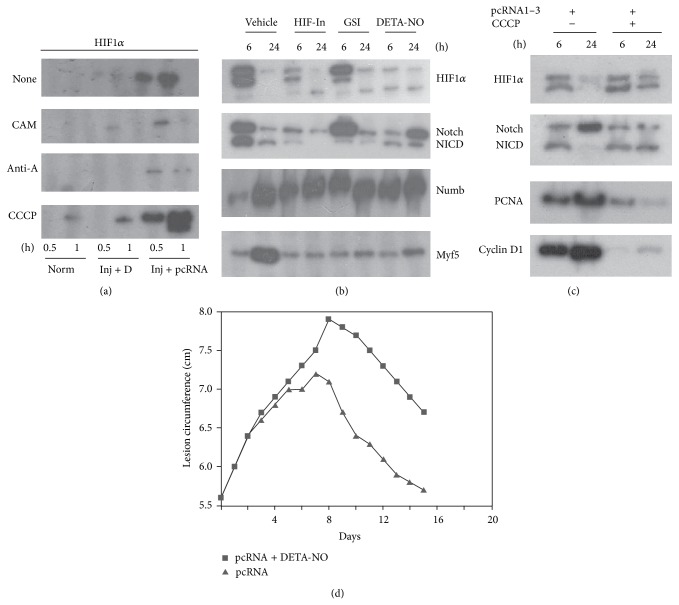
Effect of inhibitors on the hypoxic response and myogenesis. Injured muscle was treated with pcRNA cocktail for the indicated times in presence of the indicated inhibitors. CAM, chloramphenicol; AntA, antimycin A; CCCP, m-chlorocarbonylcyanide phenylhydrazone; HIF-In, HIF inhibitor; GSI, *γ*-secretase inhibitor; DETA-NO, diethylenetriamine/nitric oxide adduct. (a–c) Western blots were probed with antibody against the indicated proteins. (d) The lesion size was measured in presence or absence of DETA-NO.

**Figure 3:**
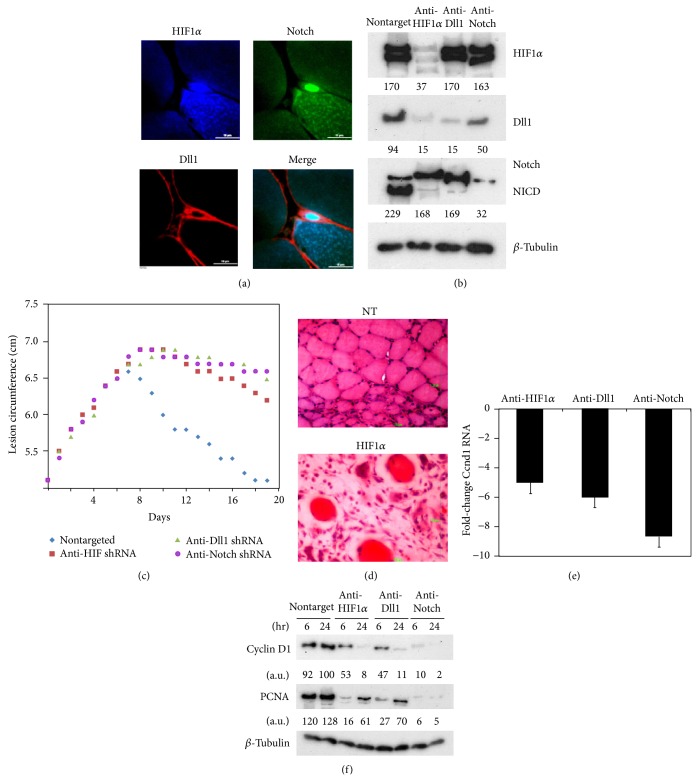
HIF-Dll-Notch axis in pcRNA-induced myogenesis. (a) A triple-stained section of injured quadriceps muscle treated with pcRNA1–3 combination for 4.5 h, showing HIF1*α* (blue) concentrated in the nucleus of an activated satellite cell, colocalizing with Notch (green) and Dll1 (red) attached to the plasma membrane as well as the plasma lemma of the underlying myofiber. Scale bar, 10 *μ*m. (b) Effect of RNA interference on protein expression in injured muscle infected for 2 d with lentivirus expressing shRNA targeted against the indicated mRNAs and then administered pcRNA1–3 for 6 or 24 h, before western analysis of indicated proteins. (c) Effect of knockdown of the indicated proteins on wound resolution. (d) Hematoxylin-eosin stain of lentivirus-infected injured muscle expressing nontargeted (NT, upper) or HIF1*α*-targeted shRNA (lower), treated with pcRNA for 2 weeks. Scale bar, 100 *μ*m. (e) Fold-change in cyclin D1 (Ccnd1) mRNA level, relative to nontargeted control, in injured muscle knocked down for the indicated protein and treated with pcRNA for 6 h, determined by Q-PCR. (f) Western blots of nontargeted or targeted muscle treated with pcRNA1–3 for 6 or 24 h, probed for Ccnd1, PCNA, or *β*-tubulin.

In cultured cells such as hepatocytes, HIF induction under hypoxia is regulated by respiratory inhibitors [17, 18] that reduce O_2_ consumption by the electron transport chain, thereby increasing availability of cytosolic O_2_ (the oxygen redistribution hypothesis). We enquired if changes in the local O_2_ concentration by respiratory inhibitors and uncouplers had any effect on wound resolution. Needle-injured muscle was treated with pcRNAs for 3 h (to allow uptake and mitochondrion targeting) and then locally injected with mitochondrial inhibitors that directly or indirectly influence O_2_ uptake. HIF*α* induction was inhibited by (1) chloramphenicol, showing the requirement of mitochondrial protein synthesis to stimulate respiration (Figure 2(a)); (2) antimycin A, an inhibitor of Complex I which abolishes state 3 respiration by blocking electron transport to O_2_ (Figure 2(a)); (3) DETA-NO, which generates NO in situ to competitively inhibit cytochrome c oxidase-mediated O_2_ consumption (Figure 2(b)); and (4) HIF-In, a pharmacological inhibitor of HIF (Figure 2(b)). In contrast, the protonophore* m*-chlorocarbonylcyanide phenylhydrazone (CCCP), which stimulates respiration through uncoupling of electron transport from oxidative phosphorylation, significantly stimulated HIF*α* levels at early times (Figure 2(a)). Furthermore, while the pcRNA-induced hypoxic response was transient, CCCP induced prolonged upregulation of HIF1*α* and Notch processing until at least 24 h (Figure 2(c)).

The respiratory inhibitors had distinct effects on regeneration. In presence of the hypoxia-inhibiting NO, the inflammation was prolonged, delaying the resolution phase, which, however, occurred at approximately the same rate as in pcRNA-treated controls (Figure 2(d)). In presence of DETA-NO or HIF-In, expression of Dll1 and of Notch was delayed; the Notch antagonist Numb was upregulated at early times, and myoblast-specific Myf5 expression was not evident after 24 h (Figure 2(b)). Thus, activation of the Notch signaling pathway is affected by inhibition of the hypoxic response.

In presence of CCCP, which induces prolonged hypoxia, the activation of SCs was affected, as evidenced by reduced expression of cyclin D1, a G1 phase marker, and of PCNA, which is expressed in the S phase (Figure 2(c)). The fraction of replicating SCs (Pax7^+^PCNA^+^) cells in the mononuclear fraction 24 h after pcRNA treatment was reduced from 70% to 15% in presence of CCCP (data not shown).

### 3.3. HIF Acts through Dll1 to Activate Notch Signaling to Induce SC Proliferation

The Notch signaling pathway has been shown to be activated early in mouse postnatal myogenesis [19]. In pcRNA-treated rat muscle, satellite cells coexpressing Notch and HIF1*α* in the nucleus and Dll1 on the plasma membrane were observed (Figure 3(a)). We studied the relationship between HIF and the Notch signaling pathway in regenerating rat muscle by target-specific RNA interference using short hairpin (sh) RNAs expressed from a lentivirus vector. In presence of shRNA targeting HIF1*α*, HIF*α* levels were reduced to 16% of that in control muscles expressing nontargeting shRNA (Figure 3(b)). In HIF*α*-targeted muscle, the level of Dll1 at 6 h was reduced to 15% of controls, and concomitant with this there was reduction in the efficiency of Notch processing to NICD from 71% to 15% of the total protein (Figure 3(b)). In contrast, a lentivirus targeting Dll1 did not affect HIF1*α* but had strong effects on Notch processing (Figure 3(b)). Downregulation of Notch did not significantly affect levels of HIF1*α* and had a 2-fold effect on the Dll1 level (Figure 3(b)).

KD of HIF1*α* in regenerating muscle had a significant effect on the rate of wound resolution, with prolonged inflammation and reduced rate of resolution; KD of Dll1 or Notch had a similar effect (Figure 3(c)). After 2 weeks of pcRNA treatment of injured muscle infected with control nontargeted lentivirus, newly formed intact fibers were observed; in contrast, in HIF1*α*-KD muscle, the injury site was filled with infiltrating mononuclear cells, including lymphocytes and neutrophils, erythrocytes, fibroblasts, and damaged myofibers (Figure 3(d)). The mRNA and protein levels of cyclin D1 were downregulated in HIF1*α*-KD muscle (Figures 3(e) and 3(f)), while the entry of cells into the S phase, as judged by the expression of PCNA, was delayed (Figure 3(f)). In presence of HIF-In, the yield of proliferating Pax7^+^ satellite cells was reduced ~3-fold in HIF-In-treated muscle (data not shown). Conversely, in injured muscle treated with recombinant Dll1 (in the absence of pcRNA), cyclin D1 transcript levels were upregulated ~5-fold after 12 h (data not shown). These results provide evidence for hypoxia-driven activation of the HIF-Delta-Notch pathway influencing SC proliferation after pcRNA-induced mitochondrial restoration.

### 3.4. Effect of DMOG-Induced Hypoxic Response

**Figure 4:**
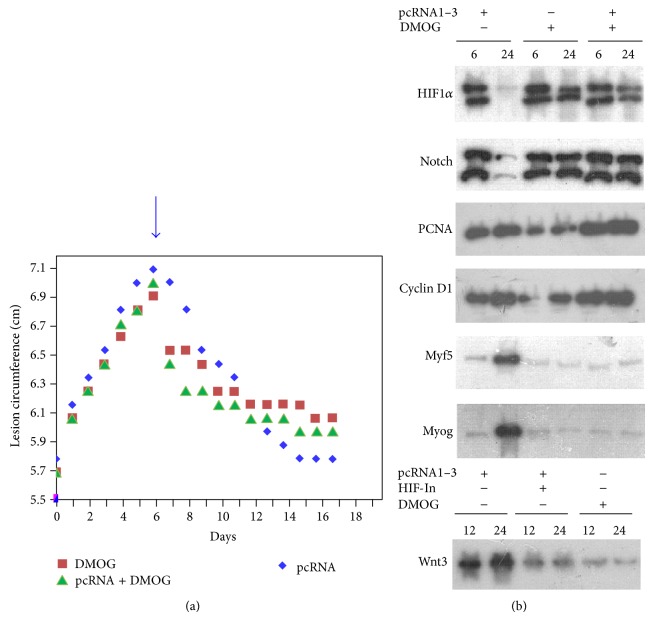
Effect of DMOG on myogenesis. (a) Lesion size of injured muscle treated with DMOG, pcRNA, or a combination of the two. (b) Effect of DMOG, pcRNA, or a combination of the two on expression of the indicated proteins after 6 or 24 h.

If activation of the HIF-Delta-Notch axis was sufficient to accelerate myogenesis, stabilization of HIF through specific inhibition of HIF prolyl hydroxylase (PHD) should effectively replace pcRNA-induced respiratory hypoxia as a wound-healing agent. In rats injected at the wound site with the PHD inhibitor dimethyloxalylglycine (DMOG [13]), there was an initial sharp lesion contraction followed by a slower rate of resolution (0.049 cm/d compared to 0.125 cm/d in pcRNA-injected muscle), with the lesion incompletely resolved after 2 weeks (Figure 4(a)). This biphasic pattern was retained in muscle treated with a combination of pcRNA and DMOG (Figure 4(a)). Western blots of DMOG-treated muscle revealed early induction of HIF1*α* with persistently high levels up to at least 24 h (Figure 4(b)). High levels of Notch/NICD were also observed at late times (Figure 4(b)). This was paralleled by high levels of PCNA and cyclin D1 expression (72% and 78%, resp., of the corresponding values in pcRNA-treated muscle at 24 h; Figure 4(b)). FACS analysis showed the presence of ~33% of Pax7^+^PCNA^+^ satellite cells in muscle after 12 h of DMOG treatment, compared to 70% in pcRNA-treated muscle (data not shown). These results indicate a high level of satellite cell proliferation in presence of DMOG. However, in contrast to pcRNA-treated muscle, there was no activation of the myogenic regulators Myf5 and Myog in DMOG-treated muscle (12% and 18%, resp., of the pcRNA-treated level at 24 h; Figure 4(b)), indicating failure of the satellite cells to differentiate. The Wnt signaling pathway is involved in switching satellite cells from proliferation to differentiation [20]; indeed, in DMOG-treated muscle, the level of Wnt3 was only 20% of that in pcRNA-treated muscle (Figure 4(b)). In presence of a combination of pcRNA and DMOG, the inhibitory effect of the latter on differentiation was maintained (Figure 4(b)).

## 4. Discussion

We show here that MR produces acute hypoxia at the injury site and that manipulating the local O_2_ concentration with inhibitors or uncouplers has specific effects on wound resolution. Our results demonstrate the importance of the microenvironment of the injury site in myogenesis.

Previous studies have documented hypoxic areas in muscle close to the incision site that return to normoxic conditions after several days [2–4]. We show that MR produces transient hypoxia in the vicinity of myofibers in which clusters of SCs accumulate (Figure 1). Transience is probably due to exhaustion at the ischemic site of carbon sources for fueling oxidative phosphorylation, thus allowing the O_2_ levels to rise quickly after a short respiratory burst. On the other hand, in intact muscle, MR induced only a gradual hypoxic response (Figure 1). This slow buildup of hypoxia in normal muscle may be due to continuous mitochondrial respiration of myofibers at a higher than normal rate exceeding the rate of O_2_ diffusion from the capillary network. In the current study hypoxia was induced by transfection of mitochondria with functional RNAs; during the normal regeneration process, acute hypoxia could be produced by involuntary exercise of the injured muscle, and/or through mitochondrial biogenesis, induced by the biogenesis factor PGC1*α* [21]. Thus, extended hypoxic conditions imposed in in vivo or in vitro studies may not reflect the true ischemic environment of the injury site.

We show that transient hypoxia and prolonged hypoxia have different effects on the regeneration process. While transient hypoxia, produced by a respiratory burst at the injury site, stimulates productive proliferation and differentiation of SCs, prolonged hypoxia leads to inhibition of differentiation. Continuous hypoxia in vivo has been reported to temporarily inhibit muscle regeneration [5]; this could be due to a delay in myoblast differentiation, as observed here. Our results are concordant with in vitro studies on myoblasts, which have shown a negative effect of prolonged hypoxia on differentiation [6]. Similarly, we have observed that in vivo, DMOG-induced prolonged hypoxia stimulates proliferation of satellite cells but blocks expression of myogenic regulators (Figure 4). It is interesting to note that while sustained hypoxia has been reported to stimulate proliferation of cloned SCs [8], it does not appear to affect overall growth of cultured myoblasts but blocks expression of MyoD, leading to accumulation of MyoD-negative cells [7]. Thus, hypoxia may specifically activate the initial SC population to form myoblasts but be dispensable for continued proliferation of these myoblasts.

Pharmacologic HIF stabilizers including DMOG, or ectopic expression of constitutionally active HIF, were found to promote angiogenesis in ischemic muscle [22] and to improve wound healing in genetically hyperglycemic mice, with evidence of enhanced migration of endothelial precursors into the wound site [23]. We found, however, that DMOG can adversely affect myogenesis by inhibiting myoblast differentiation (Figure 4). The different effects of DMOG on angiogenesis and myogenesis may be attributed to context-specific effects of Notch signaling through different combinations of Notch and Delta-Jagged ligands; for example, signaling through Jag-1 inhibits endothelial cell proliferation but promotes vascular smooth muscle cell differentiation [24].

A recent study reported that conditional cell-specific knockout (KO) of the HIF1*α* in myeloid cells resulted in reduced macrophage infiltration, fewer proliferating cells (presumably myoblasts), and delayed myogenesis, whereas KO of HIF1*α* in muscle cells had no such effects [9]. In contrast, we observed that blanket KD or inhibition of HIF1*α* at the injury site affects SC proliferation (Figure 3). The reason for the lack of effect of muscle-specific KO may be due to the fact that KO was induced by expression of the Cre recombinase from the creatine kinase (CK) promoter, which is expressed after myoblast fusion during normal development or regeneration; thus, KO would be effected only in the myofibers and not in the SCs.

In our rat quadriceps injury model, specific effects of environmental insults on the lesion size profile, with well-defined inflammatory and resolution phases (see Figures 2 and 4), were observed. We showed previously that a single dose of pcRNAs, injected in the middle of the inflammatory phase, did not influence further inflammation but specifically increased the rate of resolution; moreover, the rate of resolution was dose-dependent [10]. The electron transport inhibitor antimycin A and the uncoupler CCCP induced distinct lesion profiles, with antimycin A prolonging inflammation and CCCP producing a biphasic response, consisting of an initial rapid followed by a significantly slower resolution rate [10]. We now observe that NO, which like antimycin A reduces hypoxia though a different mechanism, produces a profile resembling that for antimycin A. On the other hand, under conditions of prolonged hypoxia, induced by either respiratory uncoupling [10] or DMOG (Figure 4), the biphasic pattern of wound resolution was obtained. This effect of DMOG persists in muscle undergoing pcRNA-mediated mitochondrial restoration (Figure 4), suggesting that hypoxia per se has an anti-inflammatory effect independent of cellular respiration and generation of ATP. Clearance of the injury site by inflammatory cells may be involved, since HIF has been shown to be involved in myeloid cell migration into the injury site [9]. Thus, besides stimulating angiogenesis, hypoxia has at least two distinct roles in wound healing: it simultaneously stimulates SC proliferation and promotes phagocyte infiltration.

## 5. Conclusions

Hypoxia is essential for myogenesis and changes in the hypoxic condition of the microenvironment of the wound site affect wound healing in specific ways. Hypoxia stimulates SC proliferation but inhibits myoblast differentiation in vivo. While transient hypoxia is beneficial, prolonged hypoxia has a detrimental effect.

## Supplementary Material

The Supplementary Material contains sequences of lentivirus shRNA inserts, primer sequences and description of antibodies used in this study.
