# Supplementary material for: Effects of Transient Hypoxia versus Prolonged Hypoxia on Satellite Cell Proliferation and Differentiation In Vivo
Source: Stem Cells Int. 2015 Feb 18;2015:961307. doi: 10.1155/2015/961307 (PMC4348605; doi:10.1155/2015/961307)
Supplement: Supplementary file 1 — The Supplementary Material contains sequences of lentivirus shRNA inserts, primer sequences and description of antibodies used in this study. [file 961307.f1.pdf]

Effects of transient versus prolonged hypoxia on satellite cell proliferation and differentiation in vivo

Sukanta Jash[1] and Samit Adhya[*]

Supplementary Information

Table S1. Lentivirus shRNA inserts

Target	shRNA Insert (sense strand)	ORF position
	Linker / sense / D loop / antisense / Linker	
Hypoxia inducible factor subunit 1 alpha	5'- CCGGGCTCACCATCAGTTACTTACGCGAACGTAAGTAACTGATGGTGAGCTTTTTG -3'<br>5'- CCGGGCTACAAGAAACCGCCTATGACGAATCATAGGCGGTTTCTTGTAGCTTTTTG -3'<br>5'- CCGGCACCGCAGACCCAGTTACAGAAACCCGAAGGTTTCTGTAACTGGGTCTGCTTTTTG -3'	209<br>689<br>1823
Delta like ligand 1 (Dll1)	5'- CCGGGCGATGAATGCATCCGATACCAACGGGTATCGGATGCATTCATCGCTTTTTG -3'<br>5'- CCGGGGGCTACACAGATCAAGAACAGAGATGTTCTTGATCTGTGTAGCCCTTTTTG -3'<br>5'- CCGGGCTGACAAGAGCAGCTTTAAGCGAACTTAAAGCTGCTCTTGTCAGCTTTTTG -3'	1047<br>2088<br>2144
Notch 1	5'- CCGGGCGTGTGCACAGAAGGTTACACGAATGTAACCTTCTGTGCACACGCTTTTTG -3'<br>5'- CCGGGGACAAACTGTGACATCAACAAACGTGTTGATGTCACAGTTTGTCCTTTTTG -3'<br>5'- CCGGGCAGCCACAGAACTTACAAATAACGATTTGTAAGTTCTGTGGCTGCTTTTTG -3'	1895<br>2480<br>7362

Table S2. Antibodies

Antigen	Specificity	Source	Vendor	Dilution used				
				WB	Confocal	ChIP	IP	
COII		rabbit	Abcam	2000	500			
COIV		rabbit	Cell Signalling		200			
Pax7		rabbit	Abcam	500	200			
HIF1α		mouse	Abcam	200	100			
HIF2α		mouse	Santacruz biotech	200				
Dll1		mouse	Santacruz biotech	200	100			
Notch1	Notch 1, NICD	rabbit	Cell Signalling	200	100			
NICD	NICD	rabbit	Cell Signalling	200	100	25		
Hey2		rabbit	BD bioscience	500				
Numb		goat	Abcam	200	100			
Myf5		mouse	BD bioscience	500	500			
MyoD		mouse	BD bioscience	1000	1000	50		
MyoG		mouse	BD bioscience	500				
Ccnd1		rabbit	Cell Signalling	1000				
PCNA		mouse	Santacruz biotech	1000	1000			

Table S3. Primers for transcript analyses.

Code	Sequence	Locus	Position	Orientation	Remarks
O-357	CGCACTTTCTTTCCAGAGTCA	Ccnd1	+813	S	Coding seq, exon4-5 junction For Q RT PCR
O-358	AAGGGCTTCAATCTGTTCCTG	Ccnd1	+887	AS	
O-344	CGATATCGCTGCGCTCGTCGTCGAC	β-actin		S	Q RT- PCR control
O-345	GGCCAGGATAGAGCCACCAATCCAC	β-actin		AS	
